# Context matters: On the road to responsible biosafety technologies in synthetic biology

**DOI:** 10.15252/embr.202051227

**Published:** 2020-12-28

**Authors:** Amalia Kallergi, Enrique Asin‐Garcia, Vitor AP Martins dos Santos, Laurens Landeweerd

**Affiliations:** ^1^ Institute for Science in Society Radboud University Nijmegen The Netherlands; ^2^ Laboratory of Systems and Synthetic Biology Wageningen University & Research Wageningen The Netherlands; ^3^ LifeGlimmer GmbH Berlin Germany

**Keywords:** Synthetic Biology & Biotechnology, S&S: Economics & Business

## Abstract

Biosafety is a major challenge for developing for synthetic organisms. An early focus on application and their context could assist with the design of appropriate genetic safeguards.

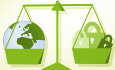

One of the goals of synthetic biology is the development of robust chassis cells for their application in medicine, agriculture, and the food, chemical and environmental industries. These cells can be streamlined by removing undesirable features and can be augmented with desirable functionalities to design an optimized organism. In a direct analogy with a car chassis, they provide the frame for different modules or “plug‐in” regulatory networks, metabolic pathways, or safety elements. In an effort to ensure a safe microbial chassis upfront, safety measures are implemented as genetic safeguards to limit risks such as unwanted cellular proliferation or horizontal gene transfer. Examples of this technology include complex genetic circuits, sophisticated metabolic dependencies (auxotrophies), and altered genomes (Schmidt & de Lorenzo, 2016; Asin‐Garcia *et al*, 2020). Much like seat belts or airbags in cars, these built‐in measures increase the safety of the chassis and of any organisms derived from it. Indeed, when it comes to safety, synthetic biology can still learn from a century‐old technology such as cars about the significance of context for the development of biosafety technologies.

Every car today has seat belts installed by default. Yet, seat belts were not always a standard component; in fact, they were not even designed for cars to begin with. The original 2‐point belts were first used in aviation and only slowly introduced for motorized vehicles. Only after some redesign, the now‐common 3‐point car seat belts would become the life‐saving equipment that they are today. A proper understanding of the context of their application was therefore one of the crucial factors for their success and wide adoption. Context matters: It provides meaning for and defines what a technological application is best suited for. What was true for seat belts may be also true for biosafety technologies such as genetic safeguards.

… when it comes to safety, synthetic biology can still learn from a century‐old technology such as cars about the significance of context for the development of biosafety technologies.

Society has a much higher awareness of technology’s risks compared to the early days of cars. Society today requires that technological risks are anticipated and assessed before an innovation or its applications are widely deployed. In addition, society increasingly demands that research and innovation take into account societal needs and values. This has led to, among others, the Responsible Research and Innovation (RRI; von Schomberg, 2013) concept that has become prominent in European science policy. In a nutshell, RRI requires that innovative products and processes align with societal needs, expectations, and values in consultation with stakeholders. RRI and similar frameworks suggest that synthetic biology must anticipate and respond not only to risks, but also to societal views that frame its evaluation and risk assessment.

## Genetic safeguards

Genetic safeguards are a technological response to societal demands for safety in synthetic biology that aim to address some of the known and unknown risks of synthetic organisms (EFSA Scientific Committee, 2020). Related to this is the Safety‐by‐Design paradigm, a risk management approach that attempts to minimize risks at the early stages of research and development (Robaey, 2018). In synthetic biology, this paradigm would mean synthetic organisms that are designed from scratch with risk management in mind. Built‐in safety by one or multiple genetic safeguards could, for example, reduce the risk that such organisms spread recombinant DNA or survive outside the conditions and environments they were designed for.

Yet, as academic and private research continues, the formulation of such genetic safeguards for real‐life scenarios remains vague. Various strategies are assumed to increase the safety of synthetic chassis and derived organisms, but discussions about their use and applicability hardly ever get more specific than that. At present, proofs of concept are being designed, developed, and evaluated irrespective of their final context of application, and contextual considerations are postponed for a later stage. This includes the necessary deliberations about utility and social desirability. We propose that an explicit strategy of contextualization, that is, an early emphasis on potential applications, can assist the development of genetic safeguards in a manner that complies with RRI. In practice, this means that important questions about genetic safeguards—their reliability, utility, or desirability—will need to be examined alongside the context of a specific application. We therefore define “context” as the final application of a “safelocked” synthetic organism; this context may be as broad as its corresponding branch of biotechnology—agriculture, medicine, or industry—or as specific as the use of one organism at a given moment and location. However, our definition does not refer to the biological context in which a safeguard strategy is implemented.

… an explicit strategy of contextualization, that is, an early emphasis on potential applications, can assist the development of genetic safeguards in a manner that complies with RRI.

## Which context?

Genetic safeguards are biocontainment and control mechanisms that would be theoretically applicable to a variety of host cells, irrespectively of their application. For example, a synthetic phosphite auxotrophy has been successfully implemented in both *Escherichia coli* and cyanobacteria to drastically reduce the risk that these cells could escape and survive in an environment without phosphite (Motomura *et al*, 2018). Another example is genome recoding, which prevents horizontal gene transfer and which has been fully or partially achieved in *E. coli*, *Salmonella typhimurium* and yeast (Kuo *et al*, 2018). Such genetic safeguards are what Mampuys and Brom (2018) describe as “horizontal integration” of biotechnology, namely the development of techniques that are increasingly more versatile and less application‐specific.

Genetic safeguards are designed as “plug‐ins”, that can be implemented in different chassis. In reality though, they are far less universally applicable than this vision would suggest. To begin with, current laboratory practice still develops genetic safeguards in and for specific organisms, with *E. coli* and yeast being the dominant ones. The effort required to implement an existing strategy in a new species is not neglectable. For instance, the genome editing tools to recode the genome are much less developed in *S. typhimurium* or other bacteria than they are in *E. coli*. Second, different chassis are not interchangeable owing to the physiological particularities of each species. For example, the engineering required for a synthetic phosphite auxotrophy depends on the specific phosphorus transport system of the species, which might consist of different transporters and transport complexes. Moreover, specific species are more suitable for certain scenarios or environments: soil bacteria, gut microbiota, or phototrophic organisms. Correspondingly, it is reasonable to expect that application contexts will dictate the use of a specific biological chassis (de Lorenzo *et al*, 2021).

There is a wide range of potential applications of synthetic biology, from medical to industrial to agricultural and environmental, that would benefit from genetic safeguards. Nonetheless, most gains are expected in novel and pervasive applications that are currently considered too risky, such as medical applications for human use or uncontained environmental applications for bioremediation (Moe‐Behrens *et al*, 2013; Schmidt & de Lorenzo, 2016). Clearly, such applications vary greatly in their characteristics, from their deployment site and ecological scale of intervention to their perceived benefits and the human practices they will affect.

Given the diversity of scenarios in which genetic safeguards may be applicable, it is striking that they are designed, evaluated, and regulated independent of their context. What is more, it is highly unlikely that the same strategies or combinations thereof will be equally useful, relevant, or desirable across such grossly diverse contexts. After all, seat belts in cars are distinctively different from seat belts in racing cars, airplanes, or rollercoasters. The aforementioned phosphite auxotrophy, for example, makes perfect sense as a biocontainment strategy for agricultural applications but may not be the best choice in biomedical settings. As we are about to see, contextualization offers tangible benefits to capture and respond to the particularities of each context and the corresponding needs and interests of stakeholders.

## Representation requires context

**Figure 1:**
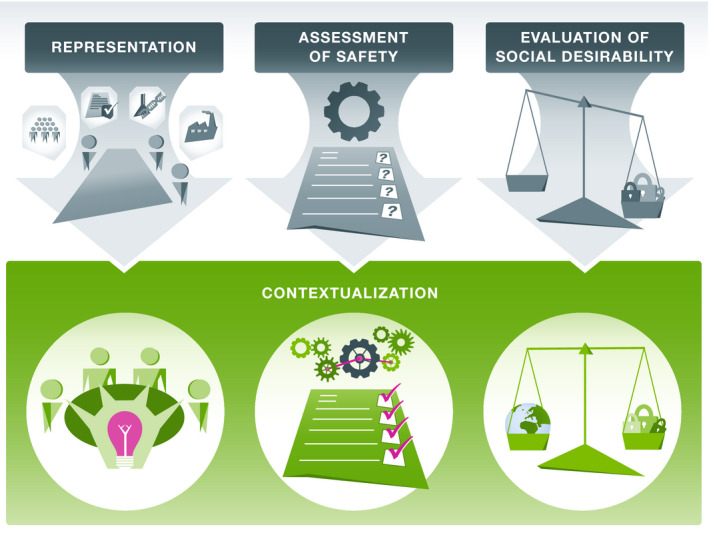
Practical benefits of contextualization for a responsible development of genetic safeguards.

The development of biosafety technologies such as genetic safeguards raises several scientific, ethical, legal, and societal questions that necessitate further research and deliberation. We suggest that the efforts needed to answer these will be both technically easier and qualitatively richer if they are organized around a specific context. Specifically, contextualization introduces concrete practical benefits both for research and for multi‐stakeholder dialogue (Fig 1).

Given the diversity of scenarios in which genetic safeguards may be applicable, it is striking that they are designed, evaluated and regulated independent of their context.

RRI emphasizes the value of early stakeholder engagement but a representative and yet manageable participation is a challenging task that only becomes harder if novel technologies are discussed without any context. Furthermore, the question of whose voices are being heard and whose stakes are being considered needs to go beyond the stereotypical categories of “science”, “industry”, and “civil society” to achieve more pluralistic and nuanced representations. Obviously, different application domains will involve different primary stakeholders: While medical practitioners and patient groups should be involved in negotiating medical applications, they may not be the most relevant representatives to deliberate agricultural applications. Even within an application domain such as medicine, the diversity of cases and practices will require further delineation. For example, the experiences, insights, and needs of medical professionals and patients will be noticeably different in the case of maintaining gut health as compared to treating cancer.

Focus on context would allow project leaders and researchers to identify and involve relevant stakeholders early on. When such a focus is not yet possible, the involvement of broad societal expertise should provide feedback for relevant applications. Once a legitimate focus is established, contextualization can help to manage stakeholder analysis, to effectively distribute often scarce resources for dialogue and move beyond stereotypical generalizations. It may also allow interested societal groups to express their specific interests rather than assembling an amorphous body of “interested citizens” or “industry representatives”. In other words, contextualization establishes a common ground for a multi‐stakeholder dialogue and joint investigation of a shared problem rather than abstract views.

## Assessment of safety is contextual

Research on genetic safeguards must generate satisfactory data about the safety and efficiency of the proposed strategies. This is not a straightforward task as laboratory practice is being hampered by a lack of adequate metrics, methods, and resources (Asin‐Garcia *et al*, 2020). First, it is practically impossible to validate safeguard strategies for every conceivable condition or application setting. An early focus on context should thus allow developers to specify finite and appropriate test conditions. Second, as laboratory test conditions and corresponding metrics are often insufficient for real‐life applications, contextualization may help to better organize research efforts: to define necessary steps for future research and divide responsibilities during the transition from development to application. Finally, the problem of funding longitudinal biosafety‐related studies is not neglectable. A clearer view on applications may allow safeguard research to engage relevant stakeholders with the means to fund or conduct these studies in their respective domains of expertise. It may also allow researchers to gather support and traction from societal groups with high stakes in the given context.

As synthetic biology tests the limits of existing risk assessment protocols and risk management practices, many call for a more flexible and adaptive risk governance. That said, the paradigm of Safety‐by‐Design, to which genetic safeguards directly contribute, requires a mode of risk management that emphasizes prevention and risk minimization via appropriate design choices. In this case, the question of what constitutes a low enough risk or, equivalently, a safe enough alternative remains relevant. Obviously, this question is highly contextual. The practice of Safety‐by‐Design as an upfront risk management tool begins with an accurate and complete formulation of the problem domain, which requires contextual information about the organism, its application and its site of deployment. A “safelocked” application is likely to be evaluated in a similar manner, unless genetic safeguards evolve into a set of standardized and previously certified parts, which will partially reduce the need for a case‐by‐case assessment (de Lorenzo *et al*, 2021). Even so, it is highly unlikely that such a toolkit can be validated for every conceivable setting. Moreover, if genetic safeguards are to satisfy a generally agreed level of safety, we must decide whether this level should cater for the most demanding settings, which is bound to cause unnecessary overhead in less demanding ones, or for a minimum set of commonly shared risks, which will inevitably require additional measures for each application.

## Evaluating social desirability

A technical assessment of safety as understood in risk assessment can inform governance by providing estimates of the probable risks of a “safelocked” synthetic biology application. However, it cannot reveal much about its social desirability. In the presence of alternative, equally “safe” or equally unsafe technologies, we may opt for ones that operate sustainably or without offending core values such as justice, privacy, or equality.

Early stakeholder engagement might ask stakeholders an impossible‐to‐answer question: to formulate an opinion about the desirability of synthetic biology by and large without knowing what this technology is actually used for. Absence of application contexts leads to grand moral debates over “naturalness” or the role of technology in society; while important, such debates rarely acknowledge the risk of not acting. Furthermore, decision‐making must acknowledge that absolute safety guarantees are unattainable and that uncertainty is a distinguishing factor of every emerging technology. A view on application contexts may therefore facilitate a deliberation that takes into consideration the tension between uncertainty and a need to act. Contextualization, or premeditation thereof, should allow us to organize dialogue so that societal actors can discuss the particularities and make assessments about the ethical, social, economic, and cultural impacts of a technology *in context*. Most importantly, contextualization appears to be the only realistic approach for an assessment that is not technology‐oriented but problem‐oriented, where decisions are based on the evaluation of alternative solutions rather than the risk‐benefit evaluation of a single technological option.

## Tensions and potential caveats

While contextualization provides obvious practical benefits, one must also consider whether it could introduce new hurdles to the responsible development of genetic safeguards. What may be the risks of an early emphasis on context? And what tensions can we already anticipate?

It is possible that an early emphasis on context will favor known and accessible scenarios at the expense of bolder or hypothetical ideas.

An early emphasis on context implies, by definition, some form of selection and prioritization. As such, it raises questions about the motivations behind these choices. An early emphasis on application contexts could be misunderstood as yet another instance of overpromising or hyping synthetic biology to secure funding or societal support. Urgent application scenarios, while noble from the point of view of societal well‐being, could also be used as a means to put pressure on societal actors or to speed up regulatory reform. To counter these risks, contextualization needs to be accompanied by a reflective mindset, a scientific agenda that is *mutually* shaped and a governance that favors resilience in the form of multiple, even redundant, solutions as opposed to silver bullets. The question of “who decides?” is again morally relevant and requires that our focus and prioritization are established in mutual dialogue.

Contextualization offers useful constraints to better organize our efforts but, what may be missed by becoming too specific too early? It is possible that an early emphasis on context will favor known and accessible scenarios at the expense of bolder or hypothetical ideas. To address this, contextualization may need to be complemented by speculative exercises, such as the use of future scenarios in technology assessment, or even by collaborating with artists and designers. To prevent a preliminary technology lock‐in, contextualization should once again be understood as part of an effort to generate and evaluate multiple alternative solutions for a given problem.

How specific should a context be to be useful? What is a meaningful clustering of application settings and based on which factors should different contexts be aggregated? Developing genetic safeguards for a dedicated, strictly defined context should result in an optimal solution for said context but may also decrease their reusability. In addition, developing safeguards for highly specific contexts will be practically similar to a case‐by‐case risk assessment, which diminishes the benefits of a standardized “safelocked” chassis. This creates a tension between contextualization on the one hand, and the basic tenets of synthetic biology, namely standardization, modularity, and abstraction, on the other. Synthetic biology promotes standardization in the form of a hypothetical repertoire of standardized chassis that are applicable to multiple contexts. This further implies that interchangeable modules or “plug‐ins” created for these standardized cells should also be developed in a context‐independent manner so as to work optimally everywhere, without too much regard about the module‐chassis combination. In the case of genetic safeguards, this is rather unrealistic and some middle way is needed between the idea of universal “plug‐ins” and the customization associated with contextualization.

## Conclusion

Our interest in contextualization corresponds with an interest in developing genetic safeguards from proofs of concept into useful tools. Our proposed strategy is primarily a response to very practical considerations that hinder this process. While contextualization does not provide all the answers, it could support synthetic biologists in devising appropriate and actionable roadmaps for agreed application context.

While contextualization does not provide all the answers, it could support synthetic biologists in devising appropriate and actionable roadmaps for agreed application context.

Although it is primarily a strategy for responsible research and development, contextualization comes with implications about the future *use* of genetic safeguards as part of the risk governance of synthetic biology. It implies that a one‐size‐fits‐all level of biosafety is inappropriate, even irrelevant, and that multiple safety measures need to be customized to different contexts. Contextualization, if properly defined at the right stages, provides a pragmatic way to effectively distribute limited resources and raise the investment of affiliated stakeholders, both monetary and in terms of engagement. Next, it offers a means to improve the quality and relevance of RRI and stakeholder engagement by requiring assessments of concrete issues as opposed to abstract and de‐personalized terms and by tapping into the local knowledge of participants. As such, it could become an essential strategy for meeting the principles of RRI in the field of biosafety.

## Conflict of interest

The authors declare that they have no conflict of interest.
